# Agalsidase beta treatment slows estimated glomerular filtration rate loss in classic Fabry disease patients: results from an individual patient data meta-analysis

**DOI:** 10.1093/ckj/sfaa065

**Published:** 2020-05-22

**Authors:** Alberto Ortiz, Steve Kanters, Alaa Hamed, Pronabesh DasMahapatra, Eugene Poggio, Manish Maski, Mario Aguiar, Elvira Ponce, Jeroen P Jansen, Dieter Ayers, Rachel Goldgrub, Robert J Desnick

**Affiliations:** 1 Unidad de Diálisis, IIS-Fundación Jiménez Díaz, School of Medicine, UAM, IRSIN and REDINREN, Madrid, Spain; 2 Evidence Synthesis and Decision Modeling, Precision HEOR, Vancouver, BC, Canada; 3 Sanofi Genzyme Health Economics and Value Assessment, Genzyme, Cambridge, MA, USA; 4 Biostatistical Consulting Inc., Lexington, MA, USA; 5 Sanofi Genzyme Medical Affairs, Genzyme, Cambridge, MA, USA; 6 Evidence Synthesis and Decision Modeling, Precision HEOR, Oakland, CA, USA; 7 Department of Genetics and Genomic Sciences, Icahn School of Medicine at Mount Sinai, New York, NY, USA

**Keywords:** agalsidase beta, chronic kidney disease outcomes, classic phenotype, Fabry disease, glomerular filtration rate, individual patient data meta-analysis

## Abstract

**Background:**

Fabry disease is a rare, X-linked genetic disorder that, if untreated in patients with the Classic phenotype, often progresses to end-stage kidney disease. This meta-analysis determined the effect of agalsidase beta on loss of estimated glomerular filtration rate (eGFR) in the Classic phenotype using an expansive evidence base of individual patient-level data.

**Methods:**

The evidence base included four Sanofi-Genzyme studies and six studies from a systematic literature review. These were restricted to Classic Fabry patients meeting the eligibility criteria from Phases III and IV agalsidase beta trials, including 315 patients (161 treated). Linear regression was first used to model annual change in eGFR for each patient and the resulting annualized eGFR slopes were modelled with treatment and covariates using quantile regression. These results were then used to estimate median annualized eGFR change in agalsidase beta treated versus untreated groups.

**Results:**

Imbalances across treatment groups were found in baseline age, sex and proteinuria, but not in the use of renin–angiotensin system blockers. The adjusted model suggests that treated (agalsidase beta) patients experienced a slower median eGFR decrease [2.46 mL/min/1.73 m^2^/year slower; 95% confidence interval (CI) 0.63–4.29; P = 0.0087] than comparable untreated patients. The median eGFR decrease was 2.64 mL/min/1.73 m^2^/year slower (95% CI 0.53–4.78; P = 0.0141) in treated Classic males.

**Conclusions:**

Using an expansive evidence base and robust modelling approach, these data indicate that agalsidase beta-treated patients with the Classic phenotype conserve their renal function better than untreated patients.

## INTRODUCTION

Fabry disease is a rare, X-linked lysosomal storage disorder due to mutations in the α-galactosidase A (*GLA*) gene that results in the absent or markedly reduced activity of its encoded enzyme, α-galactosidase A (α-Gal A) [1–3]. The enzymatic defect leads to the progressive systemic accumulation of its major glycosphingolipid substrates, globotriaosylceramide (GL-3) and its deacylated derivative (lyso-GL-3, also known as lyso-Gb3), in tissues and fluids [1, 2, 4]. The progressive accumulation of these glycosphingolipids eventually causes the disease manifestations including severe organ damage that leads to early demise [5].

Clinically, there are two major subtypes: the early-onset, severe ‘Classic’ and the ‘Later-Onset’ phenotypes [1–3, 6, 7]. Affected males with the Classic phenotype have little or no functional α-Gal A enzymatic activity, marked microvascular endothelial glycosphingolipid accumulation and childhood adolescence onset of clinical manifestations including acroparesthesias, angiokeratomas, hypohydrosis, gastrointestinal symptoms and a characteristic corneal dystrophy [1, 2]. The estimated incidence is 1 in 25 000–40 000 males based on newborn screening studies [8–10]. In contrast, affected males with the Later-Onset phenotype have residual α-Gal A activity, little, if any, microvascular endothelial glycosphingolipid accumulation, and therefore lack the early manifestations of males with the Classic phenotype [11–13]. However, they progressively accumulate the glycosphingolipid substrates, especially in cardiomyocytes and podocytes, and typically develop renal and/or cardiac disease in their fourth to seventh decades of life [1, 2, 6, 11–14]. The disease is progressive and may result in kidney damage and failure, hypertrophic cardiomyopathy, strokes and shorter life expectancy. In males with the Classic phenotype, life expectancy is 16 years shorter than the general population, as opposed to 5 years shorter for affected Classic Fabry females [5, 7, 15].

Current therapeutic approaches for Fabry disease include the reduction of accumulated glycosphingolipids through enzyme replacement therapy (ERT) and, more recently, a pharmacological chaperone approved for a subset of Fabry patients with amenable mutations, along with symptomatic and palliative treatments when needed [16, 17]. Licensed ERT treatments include agalsidase alfa (Replagal; Shire), agalsidase beta (Fabrazyme; Sanofi-Genzyme) and Fabagal (agalsidase beta biosimilar; Isu-Abxis); in addition there is one oral chaperone therapy available, migalastat (Galafold; Amicus) [18]. Agalsidase beta is licensed in both the USA and Europe, while agalsidase alfa is not licensed in the USA. Fabagal is approved in South Korea.

As Fabry disease is a rare condition, studies tend to be small and are thus limited in what can be demonstrated analytically. Meta-analyses are statistical methods by which results from multiple studies are combined. As a result, a series of small- and medium-sized studies can lead to a large number of patients and provide insights into aspects of treatment and disease progression that are not otherwise possible. Best practice for meta-analyses is that the data are obtained from a systematic literature review (SLR). Such an approach ensures that there is no selection bias with respect to the studies included in the analysis. The most common form of meta-analyses combines aggregate values across studies; however, the use of individual patient-level data (IPD) provides numerous advantages, most notably the ability to determine inclusion at an individual level and the ability to adjust for patient characteristics across settings. IPD meta-analysis is considered the gold-standard of meta-analyses [19], but remains quite uncommon given that IPD are generally not available. Given that Fabry disease studies tend to be small, they are also more likely to provide IPD within publications. El Dib *et al*. [20, 21] have conducted two recent meta-analyses and identified the lack of IPD meta-analyses as a limitation to their work and an area of unmet need.

While it is known that treatment with agalsidase beta can slow the decline of estimated glomerular filtration rate (eGFR) relative to receiving no treatment [22], the quantification of this benefit is not well known. Since the licensing of agalsidase beta in 2001, on the basis of Sanofi-Genzyme’s Phase III placebo-controlled trial which included 58 Classic affected patients (56 males) [23, 24], a large body of evidence on the outcomes of treatment of Fabry disease patients has been generated, including a Phase IV placebo-controlled trial of 82 Classic patients (72 males, 88%) [25] as well as data across a variety of real-world settings. The aim of this work was to determine the long-term effect of agalsidase beta on eGFR using an expansive evidence base that combines data from four Sanofi-Genzyme studies and six published studies obtained from an SLR.

## MATERIALS AND METHODS

### Evidence base

A broad evidence base was obtained by combining four Sanofi-Genzyme studies and data from a review of published literature. Specifically, we used data from the following four Sanofi-Genzyme studies:

- AGAL-1-002-98 [23]: a Phase III placebo-controlled trial involving 58 Classic patients (56 males, 97%);
- AGAL-005-99 (NCT00074971) [24]: a Phase III open-label extension on the same 58 patients;
- AGAL-008-00 (NCT00074984) [25]: a Phase IV placebo-controlled trial on 82 Classic patients (72 males, 88%); and
- AGAL-014-01 [26]: an observational Natural History study of historical controls including 123 Classic patients (114 males, 93%) who met the eligibility criteria to either the Phase III or Phase IV trial. The study was conducted between 2001 and 2002 prior to the approval of ERTs.

**FIGURE 1:**
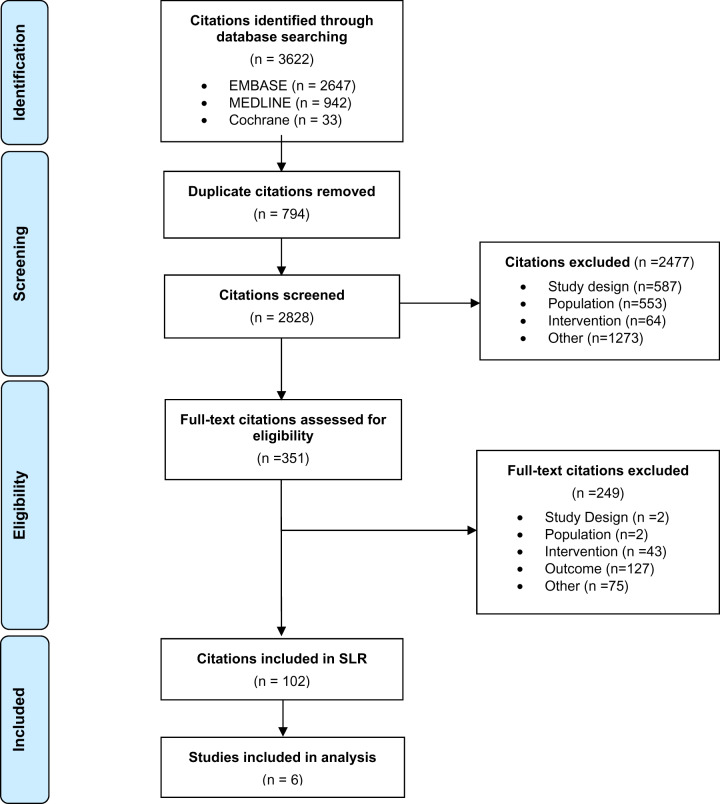
Study selection flow diagram.

The SLR included randomized clinical trials and observational studies (except case reports) that reported on patients with Fabry disease who had received agalsidase beta, placebo and/or no treatment (natural history) with outcomes data on eGFR over time. The SLR was conducted on 12 February 2018 using the databases on Ovid: Medline, Embase and the Cochrane central register of controlled trials. A total of six published studies were identified as shown in the study flow diagram (Figure 1) [27–32]. Table 1 provides a description of the six included studies. Search strategies and the table of inclusion criteria can be found in the Supplementary data, Web Appendix.

**Table 1.** List of included studies from the SLR and Sanofi-Genzyme studies

Study ID	Publications	Patients	Analyses set	Treated	Untreated	Minors	Females	Region
Breunig *et al.* [27]	Clinical benefit of ERT in Fabry disease	25	17	17	0	0	4	Germany
Politei *et al.* [28]	Fabry disease: multidisciplinary evaluation after 10 years of treatment with agalsidase beta	6	6	6	0	1	2	Argentina
Kim *et al.* [32]	Long-term ERT for Fabry disease: efficacy and unmet needs in cardiac and renal outcomes	19	15	15	0	1	4	Korea
Lin *et al.* [29]	Clinical observations on ERT in patients with Fabry disease and the switch from agalsidase beta to agalsidase alfa	9	1	1	0	0	0	Taiwan
Pisani *et al.* [30]	Effects of switching from agalsidase beta to agalsidase alfa in 10 patients with Anderson–Fabry disease	10	10	10	0	0	3	Italy
Tahir *et al.* [31]	Antiproteinuric therapy and Fabry nephropathy: sustained reduction of proteinuria in patients receiving ERT with agalsidase beta	11	6	6	0	0	2	USA
Sanofi-Genzyme studies								
AGAL-1-002-98	A Phase III placebo-controlled trial involving 58 Classic patients (56 males)	58	58	29	29	3	2	Global
AGAL-005-99 (NCT00074971)	A Phase III open-label extension on the same 58 patients	58^a^	57^a^	57^a^	0	3^a^	2^a^	Global
AGAL-008-00 (NCT00074984)	A Phase IV placebo-controlled trial on 82 Classic patients (72 males)	82	79	49	30	0	10	Global
AGAL-014-01	An observational natural history study of historical controls including 123 Classic patients (114 males) who met the eligibility criteria to either Phase III or Phase IV trials	412	123	0	123	9	9	Global

a Same patients as Phase III trial.

Using a data extraction form, two investigators independently extracted information from the materials obtained through the systematic searches. Data were reconciled to remove all discrepancies between reviewers and in case of disagreements, a third reviewer acted as an arbitrator. Some data were extracted from published graphs using the DigitizeIt software (version 15; Braunschweig, Germany). The Cochrane Collaboration’s Risk of Bias tool was used to assess risk of bias in the clinical trials [33] and the Newcastle–Ottawa Scale was used to assess the quality of observational studies (see Supplementary data, Web Appendix) [34].

### Construction of the analysis set

To ensure similarity of patients across studies, we only selected Classic males and heterozygous females who met the eligibility criteria to either Phase III or Phase IV trial [23, 25]: patients aged ≥16 years, with Classic phenotype, with baseline serum creatinine <3.0 mg/dL, not previously on a Fabry-specific treatment and no previous kidney transplant. Of note, the eligibility criteria of Phases III and IV trials differed mainly on the baseline serum creatinine levels (Phase III: <2.2 mg/dL and Phase IV: 1.2–3.0 mg/dL).

In addition to restricting this analysis to patients with the Classic phenotype, there were additional restraints and data cleaning. Patients were required to have follow-up data for a minimum of 12 weeks. Trial data for agalsidase beta were up to 5 years, and there were only 13 treated patients (all from the SLR) with data beyond 5 years. Thus, patients were restricted to the first 5 years of follow-up to have comparable data for both treatment arms.

For the SLR, all included patients had the Classic phenotype based on both the reported clinical findings and genotype/phenotype analyses according to two databases (fabry-disease.org and dbFGP.org).

On this basis, 315 unique patients were included in the analysis set: 133 treated patients, 153 untreated and 29 that were untreated for 6 months and then switched over to treatment for 4.5 years. All 58 patients from Phase III study and its extension were included. Patients in the Phase III placebo arm (*n* = 29) who were switched to agalsidase beta treatment in the extension were included in both untreated (duration of Phase III study) and treated (since the start of the extension study). One patient dropped out from the extension study and was only included in the placebo phase. For Phase IV trial, 79 of 82 patients were included, with the 3 excluded patients having <12 weeks of follow-up. For AGAL-014-01, the natural history study, 123 patients of 447 patients were included; and finally, for the six studies of the SLR [27–32], 80 patients had IPD that included eGFR and of these, 55 were included. This resulted in the total number of patients being 343. Counts on patient exclusion by reason are provided in the Supplementary data, Web Appendix.

In addition to patient selection, additional data cleaning and preparation steps were taken. Most notably, eGFR was calculated using the Chronic Kidney Disease Epidemiology Collaboration equation for all studies [35]. Proteinuria was measured in various fashions, particularly in AGAL-014-01. Proteinuria was converted to dipstick categories using the standardization method detailed in the Supplementary data, Web Appendix. Note that 85/315 patients did not report proteinuria. Further details on data cleaning are provided in the Supplementary data, Web Appendix.

### Statistical analyses

We used the summary measures approach (SMA) by which data were analysed in two steps: Step 1, an estimated patient-level rate of change in eGFR per year was obtained using linear regression on each patient individually and Step 2, the estimated slope coefficients from Step 1 were modelled using quantile regression with covariates of interest. SMA provides an approach that avoids a potentially incorrect covariance structure (i.e. such as within generalized linear mixed models) and has been shown to be robust to the underlying covariance structure among repeated observations [36].

For Step 1, the dependent variable was changed from baseline in eGFR, meaning that all patients started at the origin (0,0 coordinates of a Cartesian graph). As such, linear regression for each patient was fit with no intercept. We assumed that eGFR trajectories are linear over time, which was supported through graphical exploration. Furthermore, clinical progression of nephropathy in Fabry disease is linear in nature, as previously documented [37]. The notion of a linear trend in eGFR over time was critical to the selection of the SMA. In addition to linearity, results suggested that homoscedasticity was met (see Supplementary data, Web Appendix).

In Step 2, quantile regression was used to model the median, but supporting analyses covering the full range of quantiles were also explored. PROC QUANTREG in SAS (version 9.4) was used to conduct the analyses. Confidence intervals (CIs) were calculated using the Markov chain marginal bootstrap. The analysis also used a weighted approach given that in this particular evidence base, not all patients are equally informative with respect to the research objective.

**FIGURE 2:**
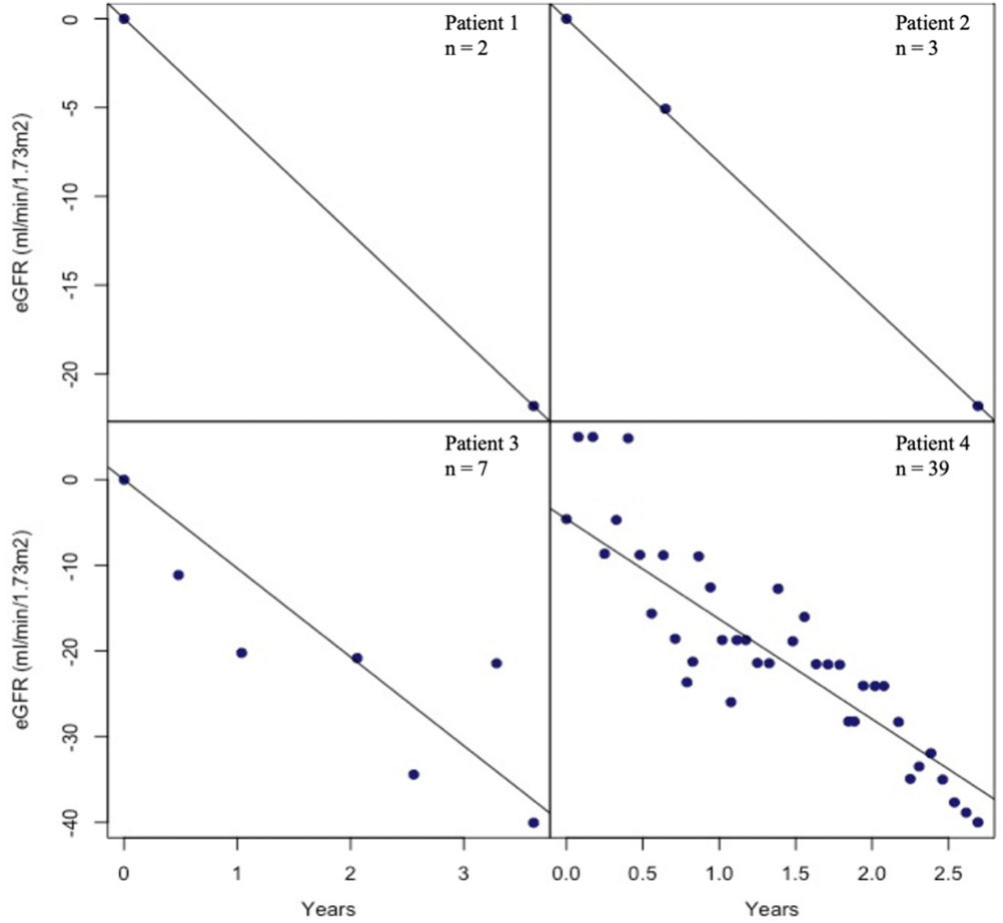
Comparing sample sizes in consideration for weights. Examples of eGFR trajectories for four patients with different number of observations. The number of observations in the overall study population varied from 2 to 41 across patients. The dependent variable was changed from baseline in eGFR, meaning that all patients started at the origin (0,0 coordinates of a Cartesian graph). The top two panels demonstrate that for patients with small number of measurements, using an inverse variance weighting would either be infeasible or be limited by overfitting. It is for this reason that the weights assuming equal residual variance across patients were favoured.

The natural choice of weights for Step 2 was the inverse variance of each slope estimate [38]. Figure 2 presents examples of eGFR trajectories for four patients. The number of observations varied from 2 to 41 across patients. There were two issues with using an inverse of estimated variance of estimated slope weight: overfitting leading to artificially small variances and undefined variances. To avoid both these issues, it was assumed that the conditional normal distribution for each patient line shares the same conditional variance parameter $\sigma^{2}$. As such, $\sum {\text{time}}_{i}^{2}$ represents an appropriate inverse-variance weighting that is proportional to the inverse variance and that avoids both issues (see Supplementary data, Web Appendix).

Covariates were chosen on the basis of known sources of heterogeneity in Fabry disease and known imbalances in the data. As such, the covariates of interest were sex, baseline age and proteinuria. For age, a threshold of 25 years was used that represents the age cut-off value for favourable early treatment effect [15]. For proteinuria, we used the dipstick categorizations with an additional category for unreported. We combined 3+ and 4+ with the 2+ category, given the small number of such patients. A further discussion on covariates is provided in the Supplementary data, Web Appendix. We approached model selection from an explanatory modelling perspective. The adjusted model included all available imbalanced known confounders. Age was deemed a potential confounder, which was tested using a change-in-estimate criterion with the conservative cut-off of 5% [39, 40].

The first set of sensitivity analyses consisted of reducing the evidence base as follows: (i) removal of Phase III trial and (ii) removal of the SLR studies. The second set of sensitivity analyses consisted of alternative covariate selection. These included the removal of females from the data and the model, the inclusion of age and including urine protein to creatinine ratio (uPCR) instead of dipstick urine as a measure of proteinuria.

## RESULTS

Baseline characteristics comparing treated and untreated patients are presented in Table 2. There were statistically significant differences between treated and untreated patients with respect to age at baseline and at diagnosis, with untreated patients being younger in both cases. With respect to sex, there was a higher proportion of females in the treatment group, and with respect to proteinuria, treated patients had a higher level of baseline proteinuria. Importantly, the proportion of individuals using angiotensin-converting enzyme inhibitors or angiotensin receptor blockers was well balanced across treatment arms. We also note that the difference in follow-up was statistically differentiable; on average, untreated patients had a shorter follow-up (difference of 3 months). Removing the placebo arm of Phase III trial led to non-significant differences in follow-up. Despite 29 untreated patients having only 6 months of follow-up, there was an acceptable number of patients in both treatment groups throughout the duration of study, with 67 treated patients and 55 untreated patients having ≥4 years of follow-up.

**Table 2.** Baseline characteristics by treatment arm

Covariate	Agalsidase beta patients (*n* = 161)	Untreated patients (*n* = 182)	P-value
Age, mean (SD), years	39.9 (11.7)	34.6 (11.6)	<0.0001
Males, %	110 (82.7)	169 (92.9)	0.0141
Caucasian, %	76 (57.1)	158 (86.8)	0.7276^a^
Black, %	1 (0.8)	3 (1.7)	
Asian, %	2 (1.5)	2 (1.1)	
Hispanic, %	11 (8.3)	15 (8.2)	
Other, %	1 (0.8)	4 (2.2)	
Missing	42 (31.6)	0 (0)	
Follow-up, mean (SD),^b^ years	2.9 (1.4)	2.6 (1.8)	0.0451
Weight, mean (SD), kg	68.8 (10.9)	70.0 (11.9)	0.7389
Age at diagnosis, mean (SD), years	33.3 (13.1)	25.2 (12.5)	<0.0001
eGFR, median; mean (SD), mL/min/1.73m^2^	85.3; 85.5 (35.4)	88.2; 88.7 (33.4)	0.4922
Serum creatinine, mean (SD), mg/dL	1.23 (0.58)	1.18 (0.48)	0.8718
Proteinuria—trace/negative, *n* (%)	45 (33.8)	77 (42.3)	<0.0001
Proteinuria—1+, *n* (%)	40 (30.1)	18 (9.9)	
Proteinuria—2+, *n* (%)	17 (12.8)	16 (8.8)	
Proteinuria—3+, *n* (%)	7 (5.3)	5 (2.8)	
Proteinuria—4+, *n* (%)	5 (3.8)	0 (0)	
Proteinuria—unreported, *n* (%)	19 (14.3)	66 (36.3)	
uPCR, mean (SD)	1.18 (1.37)	0.82 (1.20)	0.0050
Renin–angiotensin system blocker (ACEi/ARB)	29 (18.0)	31 (17.0)	0.8117
Systolic BP, mean (SD), mmHg	126.0 (16.3)	124.6 (16.6)	0.5754
Diastolic BP, mean (SD), mmHg	75.5 (10.9)	75.1 (11.2)	0.6815

a Chi-squared test omitted missing values.

b Follow-up capped at 5 years to reflect the analysis.

Wilcoxon rank sums test used for continuous variables. BP, blood pressure; ACEi, angiotensin-converting enzyme inhibitor; ARB, angiotensin receptor blockers. Percentages are column percentages.

**FIGURE 3:**
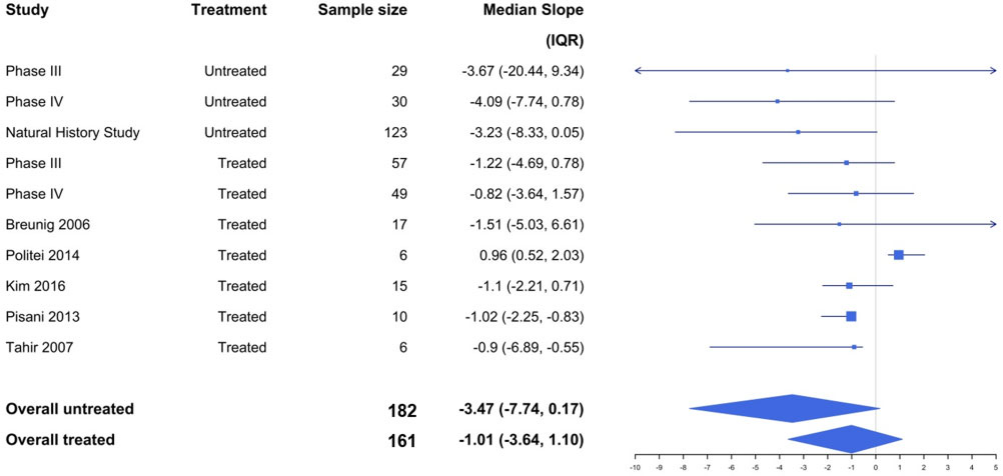
Forest plot comparing the adjusted median eGFR slopes in agalsidase beta treated versus untreated. Adjusted median eGFR slopes and interquartile range for the overall treated and untreated groups and the individual studies. After adjusting for the noted imbalances in gender and proteinuria, the treatment effect was found to be significant (P = 0.0087). The median decline in agalsidase beta-treated patients is 1.01 mL/min/1.73/m^2^/year compared with a decline of 3.47 mL/min/1.73 m^2^/year in untreated patients. Agalsidase beta-treated patients decreased by a median eGFR of 2.46 mL/min/1.73 m^2^/year (95% CI 0.63–4.29) slower than a comparable untreated patient. The study by Lin *et al.* was not shown as there was only one patient and dispersion could not be calculated.

Table 3 provides results of the principal analyses and the sensitivity analyses. According to the unadjusted model, the median eGFR loss for an untreated patient was −3.47 mL/min/1.73 m^2^ (95% CI −5.32 to −1.61) per year and for treated patients −2.43 mL/min/1.73 m^2^ (95% CI −3.53 to −1.33) per year. As a result, the difference between treated and untreated patients was 1.04 mL/min/1.73 m^2^ (95% CI −0.94 to 3.02) per year slower in treated patients. After adjusting for the noted imbalances in gender and proteinuria, the treatment effect was found to be significant (P = 0.0087). Specifically, according to the adjusted model, agalsidase beta-treated patients decreased by a median eGFR of 2.46 mL/min/1.73 m^2^/year (95% CI 0.63–4.29) slower than a comparable untreated patient. The adjusted overall treated and untreated slopes and those from the individual studies are presented as a forest plot in Figure 3.

**Table 3.** Covariate coefficient estimates (median values) from Step 2 models with 95% CIs

	Principal analyses				Study selection sensitivity analyses				Covariate selection sensitivity analyses					
Covariates	Unadjusted model		Adjusted model		Phase III removed		SLR removed		Age added		Females removed		Using uPCR	
	Estimate (95% CI)	P-value	Estimate (95% CI)	P-value	Estimate (95% CI)	P-value	Estimate (95% CI)	P-value	Estimate (95% CI)	P-value	Estimate (95% CI)	P-value	Estimate (95% CI)	P-value
Change in eGFR per year among untreated patients	**−3.47 (−5.32 to −1.61)**	**0.0003**	**−3.47 (−5.50 to −1.44)**	**0.0009**	**−3.47 (−5.98 to −0.96)**	**0.0069**	**−3.14 (−5.53 to −0.75)**	**0.0103**	**−3.47 (−5.67 to −1.27)**	**0.0021**	**−3.30 (−5.52 to −1.09)**	**0.0036**	**−3.75 (−6.72 to −0.77)**	**0.0139**
Change in eGFR per year among treated patients^a^	**−2.43 (−3.53 to −1.33)**	**<0.0001**	**−**1.01 (**−**2.69 to 0.67)	0.2362	0.17 (**−**2.48 to 2.82)	0.8991	**−**1.08 (**−**2.86to 0.70)	0.2346	**−**1.01 (**−**3.15 to 1.12)	0.3520	**−**0.66 (**−**2.21 to 0.89)	0.4030	**−**1.28 **−**3.00 to 0.44)	0.1435
Difference in change in eGFR per year for treated patients relative to untreated patients	1.04 (−0.94 to 3.02)	0.3031	**2.46 (0.63 to 4.29)**	**0.0087**	**3.64 (1.23 to 6.05)**	**0.0033**	2.06 (−0.33 to 4.45)	0.0913	2.46 (−0.07 to 4.98)	0.0568	**2.64 (0.53 to 4.78)**	**0.0141**	2.47 (−0.20 to 5.13)	0.0691
Women versus men^b^	–	–	1.38 (−0.91 to 3.68)	0.2367	−1.00 (−3.57 to 1.58)	0.4473	1.81 (−2.34 to 5.96)	0.918	1.38 (−1.47 to 4.23)	0.3400	–	–	2.65 (−3.51 to 8.81)	0.3972
Proteinuria 1+ versus trace/negativeb	–	–	−2.21 (−4.74 to 0.31)	0.0853	−1.98 (−4.71 to 0.75)	0.1537	−**3.33 (**−**6.14 to** −**0.53)**	**0.0199**	−2.21 (−4.90 to 0.47)	0.1059	–	–	–	–
Proteinuria 2–4+ versus trace/negative^b^	–	–	−**4.97 (**−**7.77 to** −**2.17)**	**0.0005**	−**6.62 (**−**10.71 to** −**2.52)**	**0.0016**	−**5.13 (**−**9.21to** −**1.04)**	**0.0141**	**-4.97 (**−**8.43 to** −**1.51)**	**0.0049**	−2.58 (−5.25 to 0.10)	0.0589	–	–
Proteinuria unreported versus trace/negative^b^	–	–	0.26 (−2.35 to 2.86)	0.8457	0.60 (−2.45 to 3.65)	0.6981	−0.07 (−3.91 to 3.76)	0.9697	0.26 (−2.40 to 2.92)	0.8489	−**5.55 (**−**8.99 to** −**2.10)**	**0.0017**	–	–
Age <25 years versus ≥25 years^b^	–	–	–	–	–	–-	–	–	0.35 (−2.28 to 2.99)	0.7924	0.09 (−2.87 to 3.06)	0.9509	–	–
uPCR^b^	–	–	–	–	–	–	–	–	–		–	–	−1.38 (−2.98 to 0.23)	0.0919

a Not a model coefficient—calculated using slope for untreated patients and effect modification term.

b Value represents the difference in change in eGFR per year between the listed groups. A positive value denotes slower loss of eGFR.

Values in bold are statistically significant at the 0.05 significance level.

Adjustments for imbalances led to a larger estimated treatment effect. Of the covariates, only proteinuria 2–4+ was statistically significant: higher levels of proteinuria were associated with steeper annual declines in eGFR, in accordance with the exploratory figures. The adjusted model had a low coefficient of determination of 0.088 (R1, as used for quantile regression).

Results of the sensitivity analyses based on study inclusion are presented in Table 3. Removing Phase III trial had the effect of increasing the magnitude of the estimated treatment effect and the treatment effect remained statistically significant (P = 0.0033). When removing the SLR studies, the estimated treatment effect was lower in magnitude, the difference in eGFR loss changed from 2.46 mL/min/1.73 m^2^/year to 2.06 mL/min/1.73 m^2^/year (P = 0.0913). Using the uPCR led to very similar results with respect to treatment effect. Note that this analysis only included the 173 patients that reported uPCRs. Adding age to the model made very little difference in the treatment effect, although the P-value was not statistically significant (P = 0.0568). The small effect size of age, its large P-value and negligible impact on the treatment estimate support the notion that it is not a confounder. Removing females from the data led to very similar results to the adjusted model [median difference treated versus untreated 2.64 mL/min/1.73 m^2^/year (95% CI 0.53–4.78; P = 0.0141)]. Thus, results of the sensitivity analyses with respect to covariate selection all led to similar estimated treatment effects.

## DISCUSSION

Using a large evidence base, this study found strong evidence supporting the beneficial effect of agalsidase beta on the decline of eGFR among Fabry disease patients with the more severe Classic phenotype. We combined data from the four major Sanofi-Genzyme studies with data from a recent, well-designed SLR, including only patients that had the Classic phenotype based on *GLA* mutation and clinical findings. To address the complex nature of the data, such as having repeat measurements on patients at varying time intervals, the analysis used robust methods so that results would not be biased by model misspecification. Finally, results of the sensitivity analyses aligned well with the principal analysis, suggesting that the results were also robust.

Agalsidase beta is recognized as a treatment that slows the progression of Fabry disease [20, 41]. Previous research has suggested that early treatment initiation can be beneficial [42]. The benefit of earlier treatment initiation can be justified in that slowing progression from a healthier state will naturally maximize the probability of staying healthier longer. The work by Waldek *et al.* [42], which reports on a Phase IV trial, argues that there is evidence of a reduced slope in eGFR decline among patients on agalsidase beta with mild to moderate renal dysfunction and that there is a lower risk of renal events. Our study finding showed a beneficial effect that was not associated with baseline age, which indicates that the slopes were constant with treatment regardless of the age at treatment initiation. Nonetheless, given that the eGFR decline in untreated patients is greater, it is projected that starting treatment earlier will delay the time to end-stage renal disease compared with patients who are left untreated. In addition, we noted that baseline proteinuria (a marker of disease progression) was significantly associated with treatment effect, which is consistent with published evidence [43, 44] indicating more favourable treatment outcomes if agalsidase beta is initiated before significant disease progression.

With respect to the sensitivity analyses, the removal of Phase III trial was based on removing patients who had higher eGFR values (often >120 mL/min/1.73 m^2^) that are more prone to measurement errors. Nonetheless, results for the sensitivity analyses were similar to the results of the principal analysis, which suggests robust results. This is particularly of interest for the analysis using the uPCR, given that this can be considered a better assessment of proteinuria than the dipstick measurements that allowed us to keep the whole sample within the analysis. The analysis of males only is justified as disease severity differs between sexes with the Classic phenotype, as we have observed an imbalance in sex in the data. Thus, sex is definitely a confounder to the effect of treatment on the annualized change in eGFR. With respect to age, while Arends *et al*. [15] have shown an association between age and globotriaosylsphingosine levels in men with Classic Fabry disease, it is unclear from this study whether age has an effect on the rate of change in eGFR. Graphical and numerical exploration did not suggest an association between age and annualized eGFR slope. When adding age to the model, it was not significant, thus supporting its exclusion from the principal analysis.

This study has some limitations. Key among them is the limited variability explained by the model. Heterogeneity in annualized eGFR change was present across all studies and no model was found to explain its underlying source. Another limitation was the statistical confounding between study and treatment given the non-comparative nature of some included studies. As a result, a random effect term to account for study was not feasible. By reducing the first analysis step to having no intercept, we reduced the need for a random intercept, but this still limited our ability to account for study-level clustering. Nonetheless, the IPD helped mitigate the ill-effects of lack of randomization by allowing for adjustments of observed confounders at the individual level. Additionally, reporting of patient characteristics in the IPD from the SLR evidence base was limited, so we could not control for many covariates. Nonetheless, we used covariates that are generally accepted as potential confounders of treatment effect in Fabry disease. Moreover, the conversion of quantitative values of proteinuria to the semi-quantitative dipstick categories, although computed based on standardized methods, is not optimal because the dipstick is measuring total proteins and may not be associated with microalbuminuria. To account for this, we conducted sensitivity analyses using uPCR (as available), which appeared to be consistent with the primary analysis. Finally, the study combined available IPD data from disparate sources: clinical trials and observational studies. Patients in the untreated group were primarily from the natural history study (AGAL-014-01), which was conducted prior to the approval of ERTs with a shorter follow-up period than the treated group (2.6 versus 2.9 years). To address this issue of similarity across studies, the data were restricted to patients who met the eligibility criteria of the clinical trials, and to ensure similarity of the studied groups, analyses were adjusted for potential confounders.

The results have biological plausibility. Due to their long lives and absence of mitosis, podocytes are non-renewable and accumulate large amounts of glycolipids, and evidence of podocyte injury leading to podocyte loss, glomerulosclerosis, a reduced number of nephrons and pathological albuminuria has been observed from childhood [45, 46]. Furthermore, lyso-Gb3 at concentrations found in the circulation of Fabry patients have been shown to injure podocytes, eliciting a human podocyte stress response very similar to the one elicited by high glucose levels in diabetes, which also causes a proteinuric nephropathy [47, 48]. In this regard, agalsidase beta administered at 1.0 mg/kg every other week was shown to reduce and even clear podocyte glycolipid deposits, as well as markedly decreasing circulating lyso-Gb3 levels [49].

In conclusion, using an expansive evidence base and a robust modelling approach, our analyses suggest that patients with the Classic phenotype on agalsidase beta conserve their renal function better than untreated patients and that treatment initiation will importantly slow progression towards end-stage kidney disease.

## SUPPLEMENTARY DATA

Supplementary data are available at ckj online.

## Supplementary Material

sfaa065_supplementary_data
