# Supplementary material for: Agalsidase beta treatment slows estimated glomerular filtration rate loss in classic Fabry disease patients: results from an individual patient data meta-analysis
Source: Clin Kidney J. 2020 May 22;14(4):1136–46. doi: 10.1093/ckj/sfaa065 (PMC8023189; doi:10.1093/ckj/sfaa065)
Supplement: sfaa065_supplementary_data [file sfaa065_supplementary_data.docx]

| Agalsidase Beta Treatment Slows eGFR Loss in Classic Fabry Disease Patients: Results from an Individual Patient Data Meta-Analysis |
| --- |
| *Web Appendix* |

# Contents

[Contents 2](#_Toc532400203)

[Tables 4](#_Toc532400204)

[Figures 5](#_Toc532400205)

[1 Additional Methods 6](#_Toc532400206)

[1.1 Systematic literature review 6](#_Toc532400207)

[1.1.1 Study selection 8](#_Toc532400208)

[1.1.2 Data extraction 9](#_Toc532400209)

[1.2 Risk of bias and quality assessment 11](#_Toc532400210)

[1.3 Construction of the analysis set 14](#_Toc532400211)

[1.4 Statistical analyses 17](#_Toc532400212)

[2 Additional Results 22](#_Toc532400213)

[2.1 Verifying homoscedasticity 24](#_Toc532400214)

[2.2 Univariate analyses 25](#_Toc532400215)

[2.3 Full analysis 26](#_Toc532400216)

# Tables

[Table 1: Study selection criteria to identify trials for the systematic literature review 5](#_Toc532400184)

[Table 2: Search strategy for EMBASE; EMBASE 1974 to 2018 February 11; Search executed: 12 February, 2018 6](#_Toc532400185)

[Table 3: Search strategy for MEDLINE; Ovid MEDLINE(R) In-Process & Other Non-Indexed Citations, Ovid MEDLINE(R) Daily and Ovid MEDLINE(R) 1946 to Present; Search executed: 12 February, 2018 6](#_Toc532400186)

[Table 4: Search strategy for Cochrane Register of Controlled Trials; EBM Reviews - Cochrane Central Register of Controlled Trials January 2018; Search executed: 12 February, 2018 7](#_Toc532400187)

[Table 5: Cochrane risk of bias assessment tool 10](#_Toc532400188)

[Table 6: Newcastle-Ottawa quality assessment scale – case-control studies 11](#_Toc532400189)

[Table 7: Newcastle-Ottawa quality assessment scale – cohort studies 12](#_Toc532400190)

[Table 8: Overview of patient counts by study 14](#_Toc532400191)

[Table 9: Distribution of weights by treatment arm 18](#_Toc532400192)

[Table 10: Newcastle-Ottawa quality assessment of SLR studies included in the analysis 22](#_Toc532400193)

[Table 11: Univariate analysis of annualized median slopes from step 1 24](#_Toc532400194)

# Figures

[Figure 1: Comparing sample sizes in consideration for weights 18](#_Toc532400264)

[Figure 2: Annualized slopes in relation to baseline age 21](#_Toc532400265)

[Figure 3: Annualized slopes in relation to baseline proteinuria (dipstick) 21](#_Toc532400266)

[Figure 4: Study selection flow diagram 22](#_Toc532400267)

[Figure 5: Residual plot 24](#_Toc532400268)

[Figure 6: Histogram of standardized residuals 26](#_Toc532400269)

[Figure 7: Quantile Processes with 95% Confidence Bands 27](#_Toc532400270)

# Additional Methods

## Systematic literature review

**Table 1: Study selection criteria to identify trials for the systematic literature review**

| Criteria | Description | |
| --- | --- | --- |
| **Population** | - Patients with Fabry disease | |
| **Interventions** | - Agalsidase beta | |
| **Comparators** | - Natural history and placebo | |
| **Outcomes** | Cardiac events   - Heart failure - Acute myocardial infarction (AMI), worsening ischemic disease - Symptomatic arrhythmia - CV interventions   Central nervous system (CNS) events   - Stroke - Transient ischemic attack (TIA)   Renal events   - Chronic dialysis - Kidney transplant - 33-50% Increase serum creatinine   All-cause mortality | Cardiovascular markers   - Left ventricular mass index (LVMI) - Maximal wall thickness - Interventricular septal thickness - Left-ventricular end-diastolic diameter   Renal Markers   - Estimated glomerular filtration rate (eGFR) decline over time   Disease severity and quality of life   - Mainz Severity Score Index (MSSI) - Disease Severity Scoring System (DS3) - SF-36 - Brief pain inventory |
| **Study design** | - Randomized clinical trials (RCTs) - Observational studies (except case reports) | |
| **Language** | - Only studies published in English | |

**Table 2: Search strategy for EMBASE; EMBASE 1974 to 2018 February 11; Search executed: 12 February, 2018**

| No. | Strings | Hits |
| --- | --- | --- |
| 1 | exp Fabry disease/ | 6264 |
| 2 | (fabrys disease or Anderson fabry disease or fabry Anderson disease or fabry syndrome or fabry dyslipidosis or angiokeratoma or alpha galactosidase deficiency syndrome).mp. | 2332 |
| 3 | or/1-2 | 6973 |
| 4 | exp agalsidase beta/ | 955 |
| 5 | exp agalsidase alpha/ | 838 |
| 6 | agalsidase.mp. | 1306 |
| 7 | (fabrazyme or replagal or agalsidase or (agalsidase adj3 (alpha or alfa or beta))).mp. | 1313 |
| 8 | or/4-7 | 1314 |
| 9 | (prognos* or outcome* or follow-up or predict*).mp. | 5398645 |
| 10 | exp disease progression/ | 2822987 |
| 11 | ((natural* or disease*) adj (progress* or course* or history)).mp. | 515065 |
| 12 | exp mortality/ | 912066 |
| 13 | (incidence or follow-up stud*).mp. | 1079592 |
| 14 | or/9-13 | 7586938 |
| 15 | 3 and (8 or 14) | 2897 |
| 16 | Limit 15 to english language | 2647 |

Table 3: Search strategy for MEDLINE; Ovid MEDLINE(R) In-Process & Other Non-Indexed Citations, Ovid MEDLINE(R) Daily and Ovid MEDLINE(R) 1946 to Present; Search executed: 12 February, 2018

| No. | Strings | Hits |
| --- | --- | --- |
| 1 | exp Fabry disease/ | 3082 |
| 2 | (fabrys disease or Anderson fabry disease or fabry Anderson disease or fabry syndrome or fabry dyslipidosis or angiokeratoma or alpha galactosidase deficiency syndrome).mp. | 2049 |
| 3 | or/1-2 | 3993 |
| 4 | agalsidase beta.mp. | 268 |
| 5 | agalsidase alpha.mp. | 52 |
| 6 | agalsidase.mp. | 408 |
| 7 | (fabrazyme or replagal or agalsidase or (agalsidase adj3 (alpha or alfa or beta))).mp. | 418 |
| 8 | or/4-7 | 418 |
| 9 | (prognos* or outcome* or follow-up or predict*).mp. | 3981844 |
| 10 | exp disease progression/ | 150620 |
| 11 | ((natural* or disease*) adj (progress* or course* or history)).mp. | 239984 |
| 12 | exp mortality/ | 336902 |
| 13 | (incidence or follow-up stud*).mp. | 1268631 |
| 14 | or/9-13 | 4646693 |
| 15 | 3 and (8 or 14) | 1084 |
| 16 | Limit 15 to english language | 942 |

Table 4: Search strategy for Cochrane Register of Controlled Trials; EBM Reviews - Cochrane Central Register of Controlled Trials January 2018; Search executed: 12 February, 2018

| No. | Strings | Hits |
| --- | --- | --- |
| 1 | exp Fabry disease/ | 43 |
| 2 | (fabrys disease or Anderson fabry disease or fabry Anderson disease or fabry syndrome or fabry dyslipidosis or angiokeratoma or alpha galactosidase deficiency syndrome).mp. | 27 |
| 3 | or/1-2 | 65 |
| 4 | agalsidase beta.mp. | 34 |
| 5 | agalsidase alfa.mp. | 32 |
| 6 | agalsidase.mp. | 53 |
| 7 | (fabrazyme or replagal or agalsidase or (agalsidase adj3 (alpha or alfa or beta))).mp. | 55 |
| 8 | or/4-7 | 55 |
| 9 | (prognos* or outcome* or follow-up or predict*).mp. | 406300 |
| 10 | exp disease progression/ | 6192 |
| 11 | ((natural* or disease*) adj (progress* or course* or history)).mp. | 20332 |
| 12 | exp mortality/ | 11696 |
| 13 | (incidence or follow-up stud*).mp. | 125836 |
| 14 | or/9-13 | 449807 |
| 15 | 3 and (8 or 14) | 34 |
| 16 | limit 15 to English language | 33 |

### Study selection

Two reviewers, working independently, reviewed all abstracts and proceedings identified by the search according to the selection criteria, with the exception of outcome criteria, which were only applied during the screening of full-text publications. All studies identified as eligible during abstract screening were then screened at a full-text stage by the same two reviewers. The full-text studies identified at this stage were included for the data extraction. Following reconciliation between the two investigators, a third reviewer was included to reach consensus for any remaining discrepancies. The process of study identification and selection are summarized with a PRISMA (Preferred Reporting Items for Systematic Reviews and Meta-Analyses) flow diagram.[^6^](#_ENREF_6)

Patient and publication overlap is always a concern when working within a population defined by a rare disease. Publication overlap occurs when multiple publications pertain to the same study. In the case of an RCT, multiple publications may exist with different outcomes reported in different publications. They are relatively simple to deal with because they usually report on the same number of patients. In the case of observational studies, overlap is difficult to assess because they can differ with respect to the number of patients or may only have partial overlap and may differ with respect to time of study (e.g., one publication reporting on 2001-2005 and the other on 2008-2009). We determined overlap in multiple manners. First, if overlap was explicitly stated in text or if the study was named. Second, we used the listed centers of study and countries. If a study was unique within a country, then overlap was not a concern. If two publications reported on data from the same hospital, then we assumed patient overlap. In most cases, overlap did not lead to exclusion of publications. This only happened if an overlapping publication provided no new information relative to other publications. For example, if a secondary paper discussed outcomes provided in another paper as well as outcomes that were not of interest to this study, then it was excluded on outcomes and the other paper was favored. The favored paper was determined on sample size and duration (larger and longer preferred).

### Data extraction

Using a data extraction form, two investigators extracted data from the materials obtained through the systematic search. Data were reconciled to remove all discrepancies between reviewers and in case of disagreements, resolutions were first determined through discussions by the reviewers, followed by the use of a third reviewer acting as an arbitrator if necessary. Some data were extracted from figures using the DigitizeIt software (version 15; Braunschweig, Germany). For digital extractions, the extraction was conducted by a single reviewer and then reviewed by a second reviewer. All data were stored and managed in Microsoft Excel Workbooks.

Data to be extracted were categorized as study characteristics, patient characteristics and outcomes. A typical systematic literature review consists of aggregate data. There are single values per study for study characteristics; there are single values per arm, per study for patient characteristics (with some cases where patient characteristics are provided as single values for all arms); and single values per arm, per time point, per study for outcomes. However, this SLR was different from the typical SLR in that data were reported in the evidence base as aggregate data and as IPD. As such, we organized the data extraction tables with separate sheets for study characteristics (all studies), aggregate patient characteristics, IPD patient characteristics, aggregate outcomes, and IPD outcomes.

Finally, we chose to extract data on a per publication basis. In some cases, it may be preferred to extract data on a per study basis and combine the information from multiple publications into the extraction for a given study. But given the *partial* overlaps seen in some studies, we judged that it would be more transparent to extract each publication. To help with the transparency, each publication was given an ID and each study or grouping was also given a separate ID.

Given that we included both randomized trials and observational studies, we referred to the science that was conducted as a study. Here, the term *study* does not correspond to the article describing it. Regarding baseline study design components, we extracted the following information: Study design (e.g. randomized, retrospective, comparative, open label, etc.); Study inclusion/exclusion criteria; Active treatment duration; Follow-up period duration; Sample size at baseline and follow-up by intervention; Study location; Study time period; Number and list of study sites; Treatment switching details; Subgroup availability.

Patient characteristics were extracted at the individual level and at the summary level. For summary statistics, the mean, median, standard deviation, and range were extracted for continuous variables whenever available. For dichotomous and categorical variables, the number of patients and proportion were extracted. Data were extracted for the following variables: Treatment details; Age; Sex; Ethnicity; Weight; Age at diagnosis; Age at symptom onset; Age at treatment initiation; Disease duration; Gene mutations; Phenotype; Smoker status; Prior treatment experience; Baseline cardio-vascular (CV) events; Baseline left ventricular mass (LVM; g); Baseline left ventricular mass index (LVMI); Baseline left ventricular posterior wall thickness (LVPWT); Baseline interventricular septum thickness (IST); Baseline heart rate; Baseline systolic blood pressure (SBP); Baseline diastolic blood pressure (DBP); History of proteinuria; Baseline estimated glomerular filtration rate (eGFR) and measured GFR; Baseline chronic kidney disease; Baseline Globotriaosylceramide (GL-3/GB-3) levels; Baseline GL-3/GB-3 levels in urine; Baseline Brief Pain Inventory (quality of life) - including domains; Baseline SF-36 - including domains; Baseline EQ-5D - including domains; Baseline Disease Severity Scoring System (DS3) - including domains.

Outcomes were also extracted at the individual level and at the summary level. For summary statistics, the mean, median, standard error, standard deviation, 95% confidence intervals and interquartile ranges were extracted for continuous variables whenever available. For dichotomous and categorical variables, the number of patients and proportion were extracted. Data were extracted for the following variables: CV events; Renal events; LVM (g); LVMI; LVPWT; IST; SBP and DBP; Proteinuria; eGFR and mGFR; Chronic kidney disease; GL-3/GB-3 levels in plasma and in urine; Brief Pain Inventory; SF-36; EQ-5D; DS3.

## Risk of bias and quality assessment

Table 5: Cochrane risk of bias assessment tool

| Domain | Support for judgment | Review authors’ judgment |
| --- | --- | --- |
| ***Selection bias*** | | |
| Random sequence generation | Describe the method used to generate the allocation sequence in sufficient detail to allow an assessment of whether it should produce comparable groups. | Selection bias (biased allocation to interventions) due to inadequate generation of a randomized sequence. |
| Allocation concealment | Describe the method used to conceal the allocation sequence in sufficient detail to determine whether intervention allocations could have been foreseen in advance of, or during, enrolment. | Selection bias (biased allocation to interventions) due to inadequate concealment of allocations prior to assignment. |
| ***Performance bias*** | | |
| Blinding of participants and personnel  *Assessments should be made for each main outcome (or class of outcomes)* | Describe all measures used, if any, to blind study participants and personnel from knowledge of which intervention a participant received. Provide any information relating to whether the intended blinding was effective. | Performance bias due to knowledge of the allocated interventions by participants and personnel during the study. |
| ***Detection bias*** | | |
| Blinding of outcome assessment  *Assessments should be made for each main outcome (or class of outcomes)* | Describe all measures used, if any, to blind outcome assessors from knowledge of which intervention a participant received. Provide any information relating to whether the intended blinding was effective. | Detection bias due to knowledge of the allocated interventions by outcome assessors. |
| ***Attrition bias*** | | |
| Incomplete outcome data  *Assessments should be made for each main outcome (or class of outcomes)* | Describe the completeness of outcome data for each main outcome, including attrition and exclusions from the analysis. State whether attrition and exclusions were reported, the numbers in each intervention group (compared with total randomized participants), reasons for attrition/exclusions where reported, and any re-inclusions in analyses performed by the review authors. | Attrition bias due to amount, nature or handling of incomplete outcome data. |
| ***Reporting bias*** | | |
| Selective reporting | State how the possibility of selective outcome reporting was examined by the review authors, and what was found. | Reporting bias due to selective outcome reporting. |
| ***Other bias*** | | |
| Other sources of bias | State any important concerns about bias not addressed in the other domains in the tool. If particular questions/entries were pre-specified in the review’s protocol, responses should be provided for each question/entry. | Bias due to problems not covered elsewhere in the table. |

Table 6: Newcastle-Ottawa quality assessment scale – case-control studies

| Domain | Response |
| --- | --- |
| ***Selection*** | |
| 1. Is the case definition adequate? | 1. Yes, with independent validation* 2. Yes (e.g. record linkage or based on self-reports) 3. No description |
| 2. Representativeness of the cases | 1. Consecutive or obviously representative series of cases* 2. Potential for selection biases or not stated |
| 3. Selection of controls | 1. Community controls* 2. Hospital controls 3. No description |
| 4. Definition of controls | 1. No history of disease (endpoint)* 2. No description of source |
| ***Comparability*** | |
| 1. Comparability of cases and controls on the basis of the design or analysis | 1. Study controls for X (select the most important factor)* 2. Study controls for any additional factor (this criterion could be modified to indicate specific control for a second important factor)* |
| ***Exposure*** | |
| 1. Ascertainment of exposure | 1. Secure record (e.g. surgical records)* 2. Structured interview where blind to case/control status* 3. Interview not blinded to case/control status 4. Written self-report or medical record only 5. No description |
| 2. Same method of ascertainment for cases and controls | 1. Yes* 2. No |
| 3. Non-response rate | 1. Same rate for both groups* 2. Non-respondents described 3. Rate different and no designation |

Note: A study can be awarded a maximum of one star for each numbered item within the selection and exposure categories. A maximum of two stars can be given for comparability.

Table 7: Newcastle-Ottawa quality assessment scale – cohort studies

| Domain | Response |
| --- | --- |
| ***Selection*** | |
| 1. Representativeness of the exposed cohort | 1. Truly representative of the average _______________ (describe) in the community* 2. Somewhat representative of the average ______________ in the community* 3. Selected group of users (e.g. nurses, volunteers) 4. No description of the derivation of the cohort |
| 2. Selection of the non-exposed cohort | 1. Drawn from the same community as the exposed cohort* 2. Drawn from a different source 3. No description of the derivation of the non-exposed cohort |
| 3. Ascertainment of exposure | 1. Secure record (e.g. surgical records)* 2. Structured interview* 3. Written self-report 4. No description |
| 4. Demonstration that outcome of interest was not present at start of study | 1. Yes* 2. No |
| ***Comparability*** | |
| 1. Comparability of cohorts on the basis of the design or analysis | 1. Study controls for _____________ (select the most important factor)* 2. Study controls for any additional factor (these criteria could be modified to indicate specific control for a second important factor)* |
| ***Outcomes*** | |
| 1. Assessment of outcome | 1. Independent blind assessment* 2. Record linkage* 3. Self-report 4. No description |
| 2. Was follow-up long enough for outcomes to occur | 1. Yes (select an adequate follow up period for outcome of interest)* 2. No |
| 3. Adequacy of follow up of cohorts | 1. Complete follow up - all subjects accounted for* 2. Subjects lost to follow up unlikely to introduce bias - small number lost - >____% (select an adequate %) follow up, or description provided of those lost)* 3. Follow up rate <____% (select an adequate %) and no description of those lost 4. No statement |

Note: A study can be awarded a maximum of one star for each numbered item within the selection and outcomes categories. A maximum of two stars can be given for comparability.

## Construction of the analysis set

**Patients excluded from the analysis from the AGAL-014-01 study:**

Patients were excluded as follows:

- 25 patients only had data while being under 16 years of age. Note that some patients were below 16 when initiating within AGAL1401 but having two or more measurements at 16 years of age or higher, they were included using the age-appropriate data only.
- 174 patients were excluded for having non-classic phenotype of Fabry disease. Note that this does not include any non-classic phenotypes among the 25 underage patients.
- 3 patients only had pre-diagnosis eGFR measurements (and were removed).
- 52 patients had no follow-up data.
- 8 patients were removed for having no follow-up after the restriction to 5 years of follow-up (see next section for an explanation of why this was done).
- 5 patients were removed for having no measurement of serum creatinine below 3.0 mg/dl (Eligibility criteria for both trials).
- 4 patients were removed for having previously had kidney transplants.
- 18 patients were removed for having less than 12 weeks of follow-up. Eight had one week only and most had under four weeks.

Note: Patient data was not available for 35 out of 447 patients in the AGAL-014-01 study

**Patients found in the SLR with IDP who were excluded from the analysis:**

Reasons for exclusions were as follows:

- 3 patients in Wraith et al, 2008: < 16 years of age. Wraith et al, 2008 was fully removed as a result.
- 5 patients in Tahir et al, 2007: Prior exposure to ERT (3 Replagal and 2 Fabrazyme)
- 8 patients in Breunig et al, 2006: 3 kidney transplants, 3 non-classic phenotypes, 1 for having no follow-up data and 1 for being afflicted by IgA nephritis
- 5 patients in Lin et al, 2014: 4 non-classic phenotypes and one minor (< 16 years)
- 4 patients from Kim et al, 2016: 2 minors and 2 patients with previous kidney transplants

Table 8: Overview of patient counts by study

| **Study** | **Source** | **Patients (n)** | **Analyses set (n)** | **Treated (n)** | **Untreated (n)** | **Minors** | **Females** |
| --- | --- | --- | --- | --- | --- | --- | --- |
| AGAL-1-002-98 (Phase III) | Sanofi Genzyme | 58 | 58 | 29 | 29 | 3 | 2 |
| AGAL-005-99 (Phase III extension) | Sanofi Genzyme | 58* | 57* | 57* | 0 | 3* | 2* |
| AGAL-008-00 (Phase IV) | Sanofi Genzyme | 82 | 79 | 49 | 30 | 0 | 10 |
| AGAL-014-01 (Natural History) | Sanofi Genzyme | 412 | 123 | 0 | 123 | 9 | 9 |
| Breunig (2006) | SLR | 25 | 17 | 17 | 0 | 0 | 4 |
| Politei (2014) | SLR | 6 | 6 | 6 | 0 | 1 | 2 |
| Kim (2016) | SLR | 19 | 15 | 15 | 0 | 1 | 4 |
| Lin (2014) | SLR | 9 | 1 | 1 | 0 | 0 | 0 |
| Pisani (2013) | SLR | 10 | 10 | 10 | 0 | 0 | 3 |
| Tahir (2007) | SLR | 11 | 6 | 6 | 0 | 0 | 2 |
| **Total** |  | **632** | **315** | **161** | **182** | **14** | **36** |

- **Same patients as the Phase III trial.*

**Additional Data cleaning steps**

Proteinuria was measured in various fashions, particularly in AGAL-014-01. As it is simplest to convert concentrations and daily amounts to dipstick categories, we opted for this route. Concentrations were converted as 0-29 mg/dl as trace/negative; 30-99 mg/dl as 1+; 100-299 mg/dl as 2+; 300-1999 mg/dl as 3+; and 2000 mg/dl or more as 4+.[^17^](#_ENREF_17) For daily measurements, positive values <0.5 g/day were converted to 1+, 0.5-0.99 g/day was converted to 2+, 1-1.99 g/day was converted to 3+ and 2g or more per day was converted to 4+.[^18^](#_ENREF_18) Note that measures of 0 were considered trace/negative and in the case of the Breunig et al, 2006 study, all patients with values of <0.03 g/day were also considered trace/negative. Using an approach that categorizes proteinuria, such as this one, we were able to add a proteinuria unreported category to allow all observations to be kept in the analysis. Note that 85/315 patients did not report proteinuria.

For all studies, all observations of serum creatinine above 25 mg/dl and ≤0.3 mg/dl were excluded. The most afflicted study was the Phase III trial, which had 55 such observations (all at the lower end). These included some negative values as well, likely to be codes for missing values.

For both the Phase III and IV trials, the baseline visit was called visit 0 and first infusion occurred on visit 1. For the phase III, the median time between baseline and first infusion was 6 days (interquartile range [IQR]: 3 – 13 days) and eGFR was measured at baseline followed by visit 7, so the pre-infusion baseline data were kept as baseline. For the phase IV trial, the median time was 23 days (IQR: 19-27 days). However, eGFR data were available at the date of first infusion, so baseline was taken to be visit 1 (i.e., visit 0 was removed from the analysis set). Note that this aligns with the clinical trial report, where the following can be found on page 8:[^19^](#_ENREF_19)

*Linear trend patient slopes for longitudinal estimated GFR were estimated by means of a linear mixed model. […] These slopes were based on data values from infusion 1 onward to the Final Blinded Visit defined as the first occurrence of an event, open label therapy or exit date.*

Finally, patient 20042 in the Phase IV study was censored following kidney transplant that occurred after randomization and similarly for patient 57001 in the Natural History study.

## Statistical analyses

For the 29 AGAL-1-002-98 patients in the placebo group, these patients were considered independently in the 22 weeks off treatment and in the 4.5 years on treatment during the extension period.

**Weighting**

The natural choice of weights for Step 2 is the inverse variance of each slope estimate. This is akin to meta-analysis of aggregate measures and leads to desirable estimator properties. Specifically, the reason inverse-variance weights are a natural choice is that for linear models with observations with differing variances, weighted least squares produces minimum variance unbiased estimates.[^22^](#_ENREF_22) It helps to understand that the number of observations varied from 2 to 41. Patients with only 2-3 observations were almost all from the Natural History study and the SLR, while patients with the highest amount of observations were from the Phase IV trial.

**Figure 1** presents examples of eGFR trajectories for four patients. Naturally slopes estimated on the basis of many observations should carry more weight than those based on few observations. For example, consider the four patients whose eGFR trajectories are depicted above. These patients have a sample size of 2, 3, 7 and 39.

Clearly, the estimated slope for the patient with *n = 39* is a more precise estimate than the others and in the absence of over-fitting, the inverse of the estimated variance will be larger for this estimated slope*.* However, there were two issues with using an inverse of estimated variance of estimated slope weight: overfitting leading to artificially small variances and undefined variances. The upper right-side panel shows an example of over-fitting where the line crosses through all points almost perfectly. As a result, the estimated residual variance is very small, leading to a large weight. The upper left-side panel shows an example of a line with only two observations for there is no estimated residual variance.

To avoid both these issues, we assumed that the conditional Normal distribution for each patient line shares the same conditional variance parameter $\sigma^{2}$. Since we are using linear regression without an intercept, the true variance of the estimated slope is simply $\sigma^{2}/\sum X_{i}^{2}$. In this analysis, the denominator is the sum of time squared ($\sum X_{i}^{2}= \sum{time}_{i}^{2}$). As such, assuming a shared common variance parameter, then $\sum{time}_{i}^{2}$ represents an appropriate inverse-variance weighting that avoids both issues previously discussed (inestimable variances and over-fitting). **Figure 1** presents the sum of patient weights per treatment-arm for each study. For simplicity, the SLR has been represented as a single study. As can be seen, the sum of weights for the placebo arm in the Phase III trial (AGAL-1-002-98) is very small. This is because the follow-up among these patients was very short, at approximately 22 weeks. Having low weights for these patients was a desirable feature given that there was some concern a priori over extrapolating long-term change using shorter follow-up.

**Figure 1: Comparing sample sizes in consideration for weights**


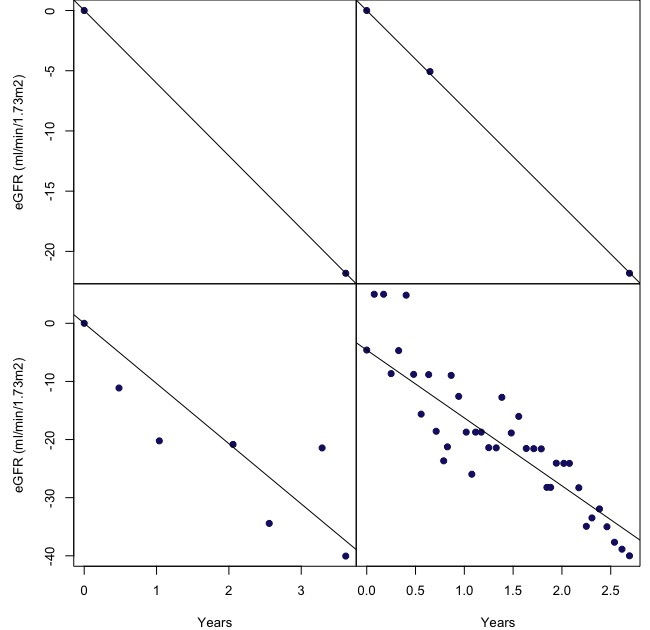


Table 9: Distribution of weights by treatment arm

| Study | Treatment | Percentage of weight by study arm |
| --- | --- | --- |
| AGAL-1-002-98 + AGAL-005-99 | Natural history | 0.1 |
|  | Fabrazyme | 33.4 |
| AGAL-008-00 | Natural history | 11.8 |
|  | Fabrazyme | 18.7 |
| AGAL-014-01 | Natural history | 26.1 |
| SLR | Fabrazyme | 9.9 |

**Covariates**

Covariates were only used in Step 2 of the modeling phase. Covariates were chosen on the basis of known sources of heterogeneity in Fabry disease and known imbalances in the data (shown in the next section). As such, the covariates of interest were sex, baseline age, and proteinuria. We also tested for interactions with baseline age, proteinuria and baseline eGFR as mentioned previously. Recall that eligibility criteria restricted all patients to classic phenotype. In addition, while use of ACE inhibitors and ARBs were considered, they were seldom reported by patients in the clinical trials (one patient consistently used them in the Phase IV trial) and they were often initiated after baseline among the modest number of patients in the Natural History study that used them. Given that we could not determine with certainty the status of concomitant ACE/ARB with observed eGFR, we opted against the use of this covariate. Moreover, we note that when it was used for the eGFR analysis in AGAL-014-01 clinical study, the ACE/ARB variable was not statistically significant.

Given that baseline age is a continuous variable, we explored whether the relationship to estimated slopes (i.e., results of step 1) were linear. We used graphical exploration and based on graphical exploration, there was no evidence of either an increasing or decreasing trend with age. **Figure 2** compares age across three age categories: 16-24, 25-44 and 45 and greater years. The 25-year threshold had been used previously,[^23^](#_ENREF_23) but for another outcome. The 45-year threshold was identified as being the threshold for the top third of observations with respect to age. As a covariate, we settled on using only the 25 year threshold given that it had been used in previous work and that there was no significant association between age > 45 years and eGFR slopes in any analyses. With respect to reference categories, male was the reference category for sex. For proteinuria we used the dipstick categorizations with an additional category for unreported. We combined 3+ and 4+ with the 2+ category, given the small number of such patients. As can be seen there was a strong association between these and slopes from Step 1 (**Figure 3**). Given the assumption that change in eGFR is linear and that each slope in Step 1 is calculated from the origin, baseline eGFR was not included in the model.

Figure 2: Annualized slopes in relation to baseline age


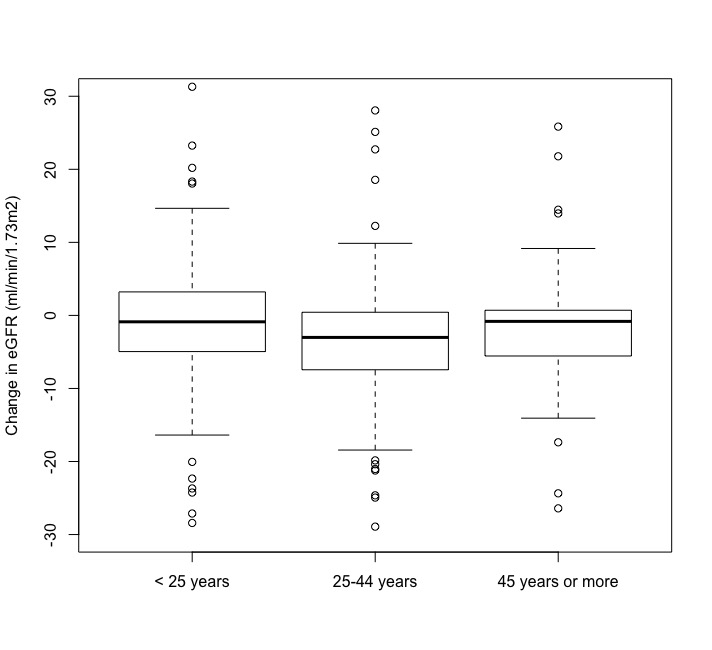


Figure 3: Annualized slopes in relation to baseline proteinuria (dipstick)

**
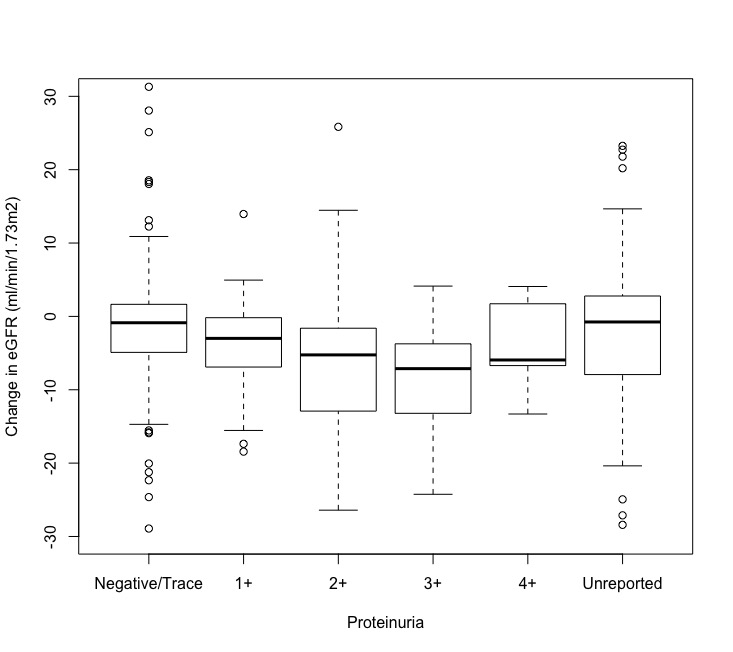
**

# Additional Results

Figure 4: Study selection flow diagram

**Screening**

**Included**

**Eligibility**

**Identification**

**Citations identified through database searching**

(n = 3622)

- EMBASE (n = 2647)
- MEDLINE (n = 942)
- Cochrane (n = 33)

**Duplicate citations removed**

(n = 794)

**Citations screened**

(n = 2828)

**Citations excluded** (n =2477)

- Study design (n=587)
- Population (n=553)
- Intervention (n=64)
- Other (n=1273)

**Full-text citations assessed for eligibility**

(n =351)

**Full-text citations excluded**

(n =249)

- Study Design (n =2)
- Population (n=2)
- Intervention (n =43)
- Outcome (n=127)
- Other (n =75)

**Citations included in SLR**

(n = 102)

**Studies included in analysis**

(n = 6)

**Table 10** provides the results of the study quality assessment conducted for the studies includes from the SLR to the analysis. Outside of being non-comparative and unblinded, there were no concerns over risk of bias.

Table 10: Newcastle-Ottawa quality assessment of SLR studies included in the analysis

| Study ID | Representativeness of the exposed cohort | Selection of the non-exposed cohort | Ascertainment of exposure | Demonstration that outcome of interest was not present at start of study | Comparability of cohorts on the basis of the design or analysis | Assessment of outcome | Was follow-up long enough for outcomes to occur | Adequacy of follow up of cohorts |
| --- | --- | --- | --- | --- | --- | --- | --- | --- |
| Breunig et al, 2006 | b | c | A | a | a, b | b | a | b |
| Politei et al, 2014 | b | c | A | a | b | b | a | a |
| Lin et al, 2014 | b | c | A | a | b | b | a | a |
| Pisani et al, 2013 | b | c | A | a | b | b | a | a |
| Tahir et al, 2007 | b | c | A | a | b | b | a | b |
| Kim et al, 2016 | a | c | A | a | a | b | a | a |

## Verifying homoscedasticity

To verify the assumptions of homoscedasticity in the models used in Step 1, we plotted the residuals from all of the regression lines fit in Step 1 of modeling against the time at which the observation was made. This is shown in **Figure 5**. Note that the variances calculated in each year are as follows: 61.8, 45.6, 57.3, 49.5, 73.13 for years 1 through 5, respectively. These do not suggest a fanning pattern in either direction and support the assumption of homoscedasticity.

Figure 5: Residual plot


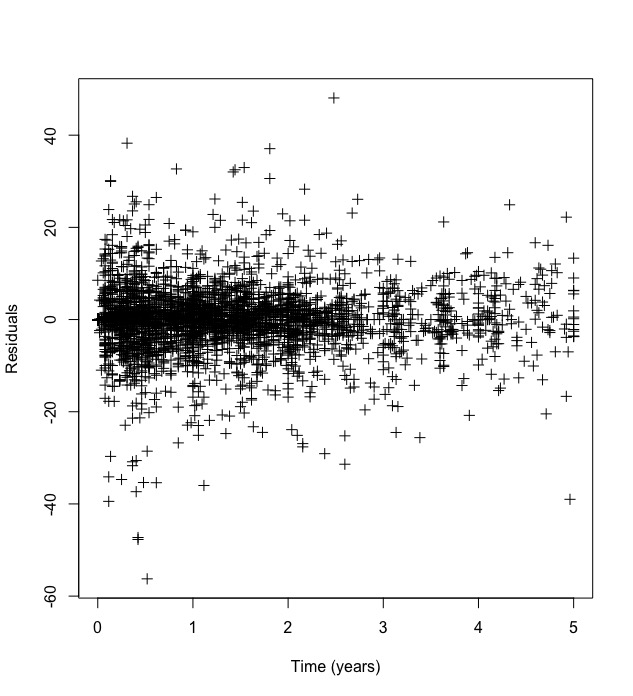


## Univariate analyses

In addition to the distribution of baseline characteristics, consider the univariate analysis of slopes obtained in the first step of analyses as presented in **Table 11**. The table presents medians and interquartile ranges (IQRs; as a single value rather than the values at either end) to better relate to the quantile regression used in step 2. The impact of using weights was most notable on the Natural History study, which shifted the median slope down and the Phase III trial, which flipped the direction of the treatment effect. Many of the positive estimated slopes within the Natural History study were based on 2 and 3 observations and the weights reduce the impact of these slopes on the median. What’s clear from **Table 11** is that using a weighted approach has a profound and, we believe, appropriate impact on results; in this case flipping the direction of the effect. While it would be preferable to have similar results using a weighted and unweighted approach, it is not the case here.

Table 11: Univariate analysis of annualized median slopes from step 1

|  | Weighted | | Unweighted | | |
| --- | --- | --- | --- | --- | --- |
| Study ID | Treated  Median (IQR) | Untreated  Median (IQR) | | Treated  Median (IQR) | Untreated  Median (IQR) |
| Phase III trial + extension | -2.30 (6.00) | -3.26 (33.77) | | -2.26 (6.07) | -1.17 (34.53) |
| Phase IV trial | -3.87 (5.81) | -4.90 (9.05) | | -3.28 (7.77) | -4.05 (7.50) |
| Natural History study | N/A | -3.47 (7.93) | | N/A | -0.91 (9.35) |
| SLR | -0.83 (2.66) | N/A | | -0.76 (4.69) | N/A |
| Overall | -2.43 (5.46) | -3.47 (8.23) | | -2.19 (6.13) | -1.25 (10.63) |

Adjustments for imbalances led to a larger estimated treatment effect. Of the covariates, only proteinuria 2-4+ was statistically significant. Higher levels of proteinuria were associated with steeper annual declines in eGFR, in accordance with the exploratory figures. The adjusted model had a low coefficient of determination of 0.088 (R1, as used for quantile regression). Thus, there remains a lot of variance that is unexplained by the model. Using a cut-off of 3 standard deviations, 30 observations (8.8%) were identified as outliers on the basis of the model residuals. Their removal had negligible impact on the coefficient of determination.

## Full analysis

The outliers explain the heavy tails in the histogram of standardized residuals presented in **Figure 6**.

Figure 6: Histogram of standardized residuals


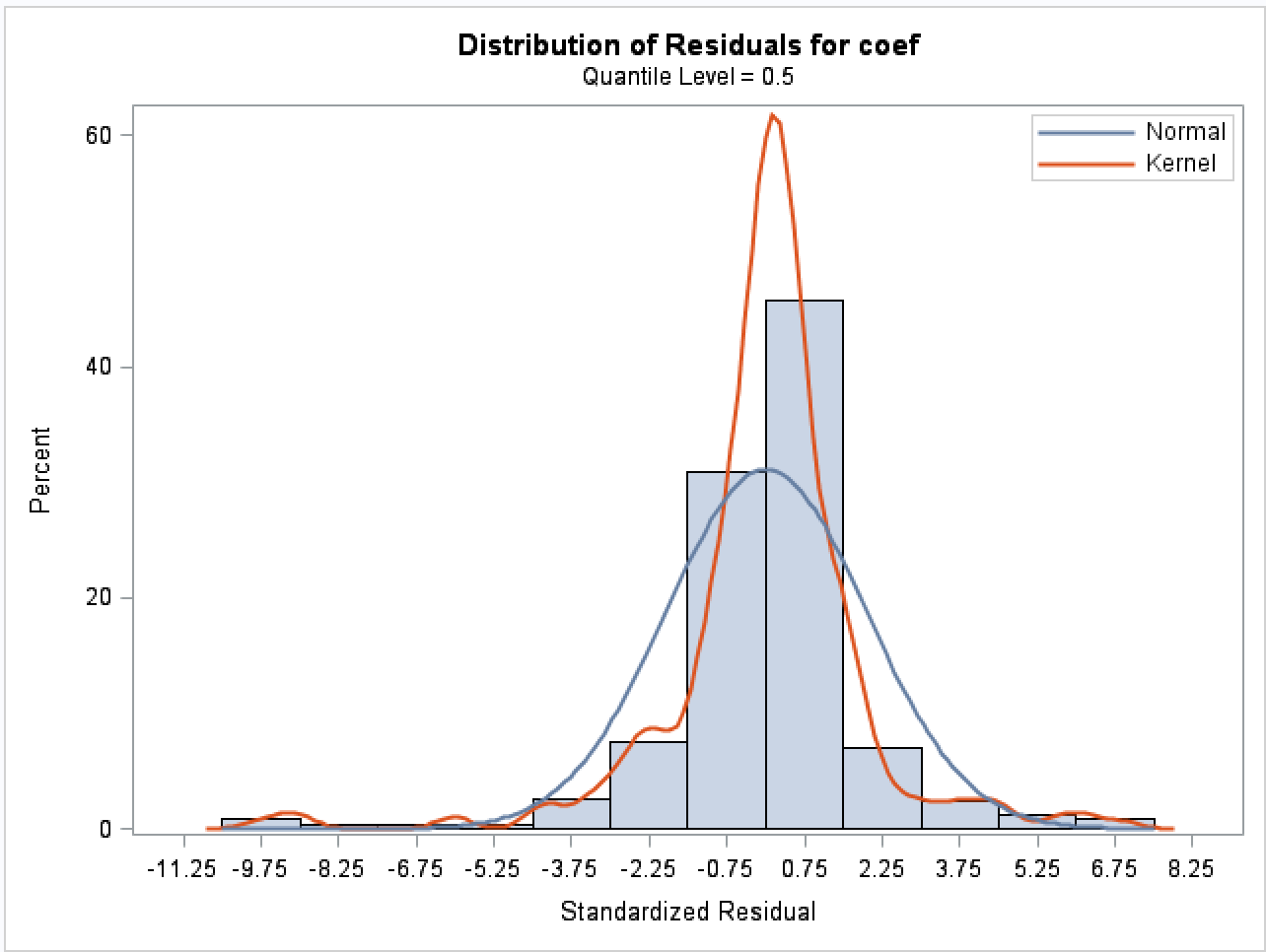


The Quantile process plots are presented in **Figure 7**. Below the 80^th^ percentile, treatment has a positive effect on eGFR (upper right-hand panel). The figure shows that from approximately the 20^th^ percentile to up to close to the 60^th^ percentile, the treatment effect is strong and statistically significant. Similarly, the effect of proteinuria appears to be constant over time. Finally, the extreme quantiles are seen to behave erratically.

Figure 7: Quantile Processes with 95% Confidence Bands


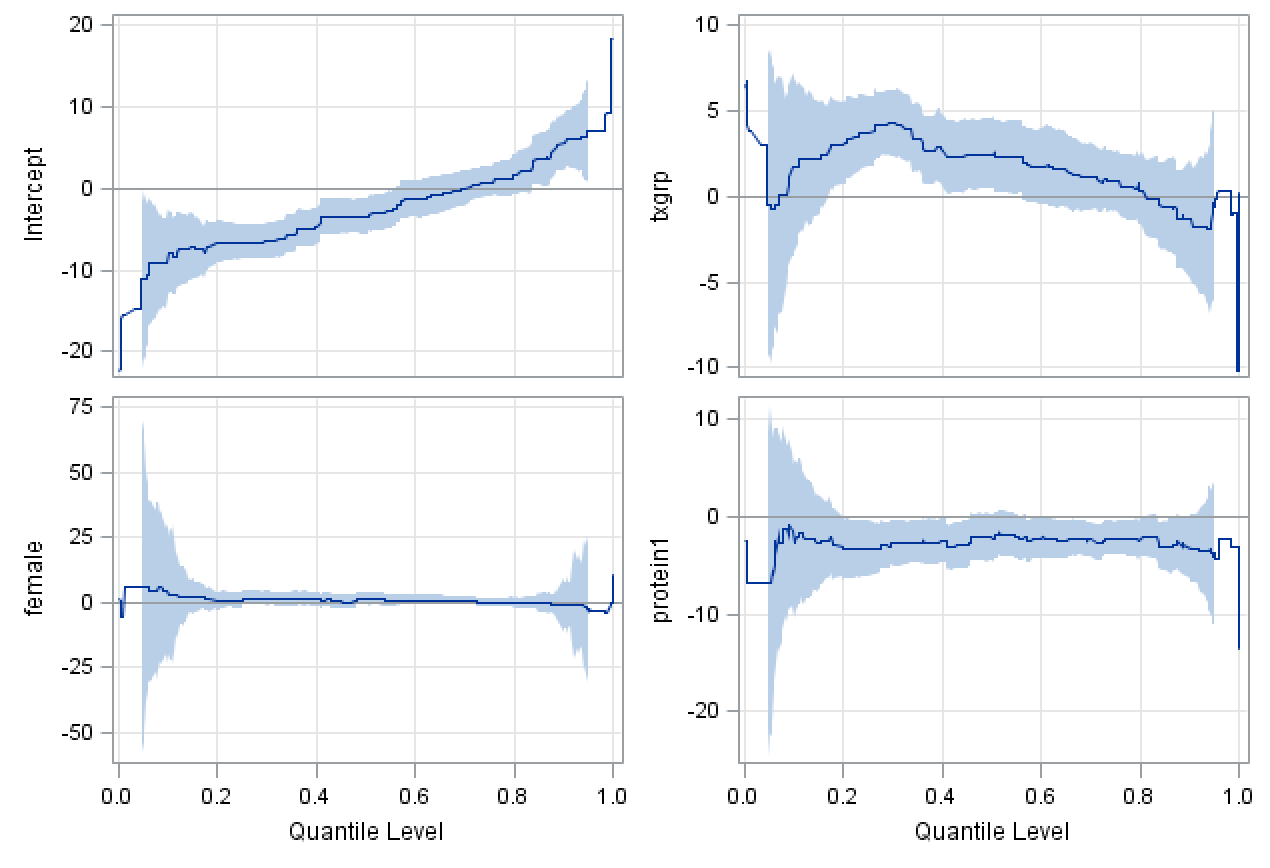


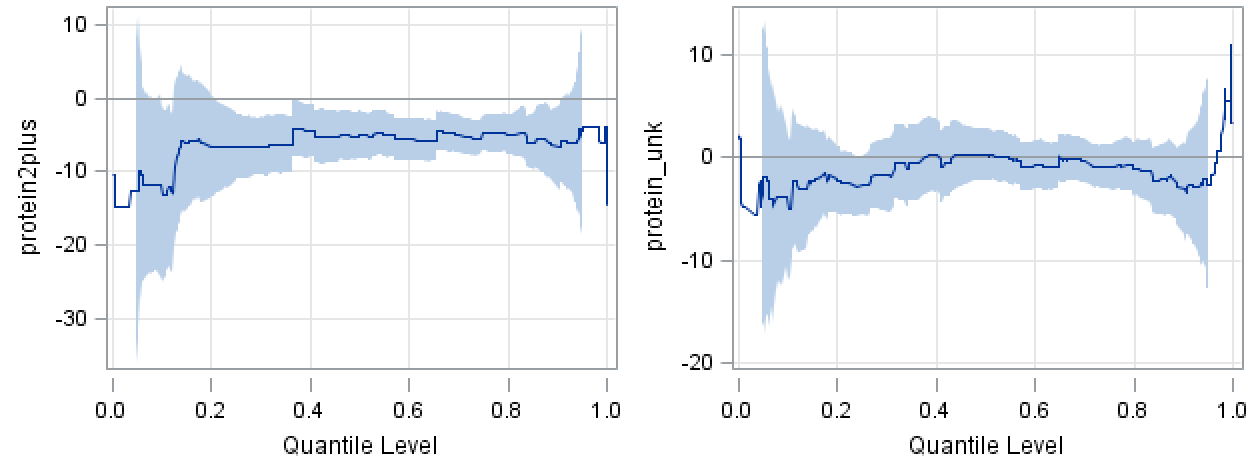


*Legend: Each figure represents the estimate of the covariate in question across the different values of the quantiles. Txgrp: Treatment group; protein1: Proteinuria 1+; protein2plus: Proteinuria 2-4+; Protein_unk: Unreported proteinuria*
